# Exploring the link between metabolic dysfunction-associated fatty liver disease and subclinical hypothyroidism in adolescents: a comprehensive review

**DOI:** 10.3389/fped.2026.1696331

**Published:** 2026-02-16

**Authors:** Xinlong Hu, Wenzai Shi, Guoshuai Xu, Wenqiang Li, Nan Yao, Guoyong Yu, Jun Qu

**Affiliations:** 1 Department of General Surgery, Aerospace Center Hospital, Beijing, China; 2 Department of Hepatobiliary Surgery, Peking University International Hospital, Beijing, China; 3 Department of Nephrology, Beijing University of Chinese Medicine Affiliated Dongzhimen Hospital, Beijing, China

**Keywords:** metabolic dysfunction-associated fatty liver disease (MAFLD), subclinical hypothyroidism (SCH), thyroid-stimulating hormone (TSH), insulin resistance, adolescents

## Abstract

**Background/objectives:**

Metabolic dysfunction–associated fatty liver disease (MAFLD) and subclinical hypothyroidism (SCH) increasingly co-occur in adolescents, yet their inter-relationship and clinical relevance remain uncertain.

**Objective:**

To synthesize evidence on epidemiologic associations, shared mechanisms, and care implications linking MAFLD and SCH in youth.

**Methods:**

We conducted a structured review of PubMed, Embase, Web of Science, and the Cochrane Library from inception to December 31, 2024, focusing on pediatric observational studies and mechanistic or interventional data relevant to adolescents. Two reviewers screened studies and extracted design, diagnostics, exposures (TSH/thyroid hormones), outcomes (steatosis severity, fibrosis, liver enzymes), and adjusted effect estimates. Risk of bias was narratively assessed for observational designs.

**Results:**

Pediatric cohorts consistently report a positive association between higher TSH (within the reference or mildly elevated range) and hepatic steatosis severity, with several studies indicating a dose–response gradient. Mechanistic evidence suggests TSHR–SREBP-1c signaling, insulin resistance, adipokine imbalance, low-grade inflammation, and gut–liver–thyroid crosstalk as plausible pathways. Adult interventional data show that levothyroxine therapy for SCH can modestly reduce liver fat and aminotransferases; however, pediatric trials are lacking. Definitions, diagnostic modalities, and confounding control vary across studies, and most pediatric evidence is cross-sectional, limiting causal inference.

**Conclusions:**

In adolescents, MAFLD and SCH appear linked through metabolic and endocrine pathways, but causality remains unproven. Risk-based screening may be warranted—thyroid testing in MAFLD and targeted liver assessment in persistent SCH—while longitudinal cohorts and pediatric trials are needed to define thresholds for intervention and potential benefits of endocrine management.

## Highlights

- Synthesizes adolescent evidence linking MAFLD and SCH and maps shared mechanisms.
- TSH may drive hepatic lipogenesis via TSHR–SREBP-1c signaling, independent of overt hypothyroidism.
- Pediatric studies show higher SCH prevalence/TSH levels with increasing steatosis severity.
- Causal mediation suggests TSH partially mediates obesity's effect on MAFLD risk in youth.
- Persistent TSH elevation associates with histologic progression; adult LT4 trials improve liver fat and enzymes, but pediatric interventional data are lacking.
- Evidence remains largely cross-sectional; priorities include longitudinal and interventional pediatric studies to establish causality and guide risk-based screening.

## Introduction

Pediatric obesity has become a global epidemic, fueling a parallel rise in metabolic and endocrine disorders among adolescents. Two conditions that frequently coexist in this population—metabolic dysfunction-associated fatty liver disease (MAFLD) and subclinical hypothyroidism (SCH)—share overlapping metabolic features and may influence each other's onset and progression (1, 2). MAFLD, a redefinition of nonalcoholic fatty liver disease (NAFLD), is diagnosed when hepatic steatosis is present alongside at least one metabolic abnormality, such as obesity, insulin resistance, or dyslipidemia. In adolescents, it is now the most common chronic liver disease, often detected incidentally during imaging or routine metabolic screening (3).

In parallel, SCH—characterized by elevated thyroid-stimulating hormone (TSH) with normal free thyroxine (FT4)—is increasingly reported in overweight and obese youth. Although sometimes regarded as benign or transient, SCH has been linked to adverse metabolic profiles, including higher body mass index (BMI), dyslipidemia, and impaired insulin sensitivity (4, 5). These abnormalities closely mirror MAFLD risk factors. Beyond association, experimental data suggest that elevated TSH may directly promote hepatic lipid deposition by stimulating hepatocyte lipogenesis, even when circulating thyroid hormone levels are normal (6, 7). Nevertheless, pediatric-specific evidence remains limited, and the direction of causality is unclear (8).

Given the long-term hepatic and cardiovascular risks of both conditions, clarifying their interplay during adolescence—a critical developmental period for metabolic programming—is clinically important. This review synthesizes current evidence on the association between SCH and MAFLD in adolescents, explores potential shared mechanisms, and identifies priorities for future clinical and translational research.

## Methods

Search strategy and selection. We searched PubMed/MEDLINE, Embase, Web of Science, and Cochrane Library using controlled vocabulary and keywords for (subclinical hypothyroidism OR TSH), (MAFLD/MASLD OR fatty liver OR steatosis), and (adolescents OR youth OR pediatric). Records were deduplicated; two reviewers independently screened titles/abstracts and full texts with adjudication by a third reviewer. A PRISMA 2020 flow diagram summarizes screening (Supplementary Figure S1). PRISMA counts reflect only pediatric primary studies included in the qualitative synthesis; adult interventional trials, mechanistic studies, and narrative reviews were cited for context and not counted as included studies.

Inclusion criteria: (i) participants ≤18 years or clearly pediatric populations; (ii) exposure: subclinical hypothyroidism (elevated TSH with normal free T4); (iii) outcome: MAFLD/MASLD or hepatic steatosis defined by imaging, biomarkers, or clinical criteria; (iv) study design: observational (cross-sectional, cohort, case-control) or trials; (v) reports association or relevant mechanistic data.

Exclusion criteria: adult-only studies; overt hypothyroidism or thyroid treatment without SCH subgroup; non-human studies unless mechanistic context only; case reports/series without comparator; abstracts lacking data; duplicate cohorts without unique analyses.

Risk of bias was assessed by two reviewers using design-appropriate tools: the Newcastle–Ottawa Scale (NOS) for case–control and cohort studies (Nichols, Untalan) and the JBI analytical cross-sectional checklist for cross-sectional studies (Choi, Kaltenbach, Xue). (Supplementary Table S1, S2); we did not dichotomize studies by arbitrary score thresholds.

## Overview of MAFLD in adolescents

MAFLD is a recently defined clinical entity that reframes hepatic steatosis by emphasizing its metabolic underpinnings. Unlike the older term nonalcoholic fatty liver disease (NAFLD), which is largely defined by excluding secondary causes, MAFLD is diagnosed when hepatic fat accumulation occurs in the presence of metabolic risk factors such as obesity, insulin resistance, dyslipidemia, or type 2 diabetes (9, 10). In pediatric patients, the diagnosis requires imaging or histological evidence of steatosis along with at least one feature of metabolic dysfunction (3). This approach better reflects the pathophysiological profile of adolescents, especially given the global rise in childhood obesity (1).

The prevalence of MAFLD in adolescents varies widely. Population-based estimates range from roughly 7% in general pediatric cohorts to over 30% in obese youth. A systematic review and meta-analysis by Anderson et al. (2015) reported a pooled prevalence of 2.6%–10% in the general pediatric population and up to 34% in obese children, reflecting the strong influence of adiposity on disease burden (1). More recent data suggest that in some clinical cohorts, particularly those using MAFLD diagnostic criteria, prevalence may exceed 50% among high-risk subgroups-such as male adolescents and individuals of Hispanic or Asian descent (11, 12). This variation reflects not only genetic predisposition but also lifestyle and environmental factors, including reduced physical activity, greater consumption of ultra-processed foods, and the growing burden of insulin resistance during puberty (13).

The pathogenesis of MAFLD is multifactorial, with hepatic insulin resistance at its core. Disrupted insulin signaling in hepatocytes promotes *de novo* lipogenesis, suppresses fatty acid oxidation, and increases the influx of free fatty acids from adipose tissue lipolysis. Together, these processes drive triglyceride accumulation within the liver. As steatosis advances, secondary insults—such as oxidative stress, endoplasmic reticulum stress, and inflammatory signaling—contribute to hepatocellular injury, nonalcoholic steatohepatitis (NASH), and ultimately fibrosis (13, 14).

Host genetics further modulate susceptibility. Variants in PNPLA3 and TM6SF2 have been linked to greater hepatic fat content and fibrosis risk in both adults and children (15, 16). The gut–liver axis has also emerged as a key player. Dysbiosis, characterized by reduced microbial diversity, increased gut permeability, and altered bile acid metabolism, may amplify systemic inflammation and accelerate hepatic lipid deposition (17, 18).

Clinically, adolescent MAFLD is often silent, detected only through elevated aminotransferase levels or incidental imaging findings. Yet even mild disease carries long-term consequences, including heightened risks of type 2 diabetes, cardiovascular disease, and progressive liver injury (18–20). Early recognition is therefore crucial—particularly in adolescents with coexisting endocrine conditions such as SCH, which may further compound metabolic and hepatic stress (21, 22).

## Overview of subclinical hypothyroidism in adolescents

SCH refers to a mild form of thyroid dysfunction in which serum TSH levels are elevated, but free thyroxine (FT4) concentrations remain within the reference range. Often asymptomatic, SCH is being recognized more frequently in adolescents, particularly in those with obesity, insulin resistance, or autoimmune thyroid disease (23, 24). The prevalence appears to rise during puberty—especially among girls—likely reflecting both physiological hormonal shifts and underlying endocrine susceptibility (25). In obese pediatric cohorts, reported rates range from 10% to 20%, compared with only 1%–4% in the general adolescent population (21).

Autoimmune thyroiditis, most commonly Hashimoto's thyroiditis, is the leading cause of SCH in this age group and is characterized by the presence of anti-thyroid peroxidase (TPO) and anti-thyroglobulin (TG) antibodies (26). Other contributors include excessive iodine intake, genetic predisposition, and hormonal fluctuations during puberty. Although FT4 levels remain normal, some adolescents may experience tissue-level hypothyroidism due to impaired conversion of thyroxine (T4) to triiodothyronine (T3) or reduced peripheral sensitivity to thyroid hormones (27).

Increasing evidence indicates that SCH, even in its subclinical form, is associated with significant metabolic alterations (28). Adolescents with SCH frequently exhibit increased body mass index (BMI), dyslipidemia characterized by elevated LDL cholesterol and total cholesterol levels, impaired glucose tolerance, and reduced insulin sensitivity. These metabolic disturbances mirror the key features of MAFLD pathogenesis, suggesting potential bidirectional interaction (29). In particular, elevated TSH—once considered a mere biomarker—may itself exert direct effects on metabolic tissues, including hepatocytes and adipocytes. TSH has been shown to influence hepatic lipid accumulation, modulate adipokine secretion, and activate inflammatory pathways, which may compound the risk of hepatic steatosis and metabolic syndrome in susceptible youth (30).

The natural course of SCH in adolescents is variable, with many cases—particularly those without thyroid autoantibodies and with TSH levels <10 mIU/L—showing spontaneous normalization over time (31–33). Treatment thresholds therefore remain debated in pediatrics. While TSH ≥10 mIU/L is often used as a pragmatic cut-off to consider levothyroxine in persistently elevated or symptomatic cases, management of milder elevations (TSH ∼4–10 mIU/L) is individualized. Decisions typically incorporate TSH trajectory across repeat measurements, clinical symptoms, goiter or thyroid autoimmunity (anti-TPO), lipid profile and insulin resistance, pubertal status, and overall cardiometabolic risk. Persistent or progressive SCH—especially in the presence of thyroid autoimmunity—is more likely to progress to overt hypothyroidism and may aggravate coexisting metabolic disturbances (34). Current pediatric guidelines do not mandate universal treatment of SCH, but rather support individualized decision-making and periodic reassessment (35). In adolescents with MAFLD, however, SCH should not be regarded as an incidental finding. Given the shared metabolic and inflammatory milieu, adolescents with SCH—particularly those with obesity or other metabolic risk factors—may warrant targeted hepatic assessment, whereas those with MAFLD and elevated TSH should receive closer endocrine evaluation. Recognizing this bidirectional overlap may help refine risk stratification and promote integrated, early interventions during this critical window of metabolic development.

## Pathophysiological connections between MAFLD and SCH

Recent evidence indicates that MAFLD and SCH in adolescents are not merely coincidental but are mechanistically linked through interconnected endocrine–metabolic pathways. Both share common risk factors—obesity, insulin resistance, and low-grade systemic inflammation—and may exacerbate one another through bidirectional feedback loops.

TSH-mediated hepatic effects appear to be a key link. Hepatocytes express TSH receptors (TSHR), and TSHR activation stimulates lipogenic transcription factors such as sterol regulatory element-binding protein-1c (SREBP-1c), enhancing triglyceride synthesis and fat accumulation (6). This signaling cascade promotes triglyceride synthesis and hepatic fat accumulation, even in euthyroid individuals (36). In adolescents with SCH, chronically elevated TSH may thus contribute directly to the pathogenesis of MAFLD, amplifying hepatic steatosis through enhanced *de novo* lipogenesis (37).

Insulin resistance serves as another major bridge. In MAFLD, impaired insulin signaling promotes increased free fatty acid flux to the liver, reduced *β*-oxidation, and triglyceride storage. SCH may worsen insulin resistance through reduced T3-mediated glucose transporter activity, mitochondrial dysfunction, and altered adipokine signaling (37). Adolescents with SCH often exhibit elevated fasting insulin and higher homeostatic model assessment for insulin resistance (HOMA-IR) scores, both of which are linked to liver fat accumulation (38).

Chronic low-grade inflammation further connects the two disorders. In MAFLD, hepatocyte injury and lipotoxicity trigger pro-inflammatory cytokine release, including tumor necrosis factor-α (TNF-α) and interleukin-6 (IL-6) (38). In SCH—particularly of autoimmune origin—systemic inflammation is also elevated, even without overt hypothyroid symptoms (39). This systemic inflammatory milieu may perpetuate hepatic dysfunction and thyroid hormone resistance, establishing a pathological feedback loop. Moreover, dysfunction of adipose tissue in both conditions contributes to an imbalance in adipokines such as leptin, adiponectin, and resistin.

Adipokine dysregulation adds another layer of interaction. In both conditions, leptin resistance can stimulate TSH secretion and hepatic lipogenesis, while low adiponectin levels reduce fatty acid oxidation and worsen insulin resistance (38, 40). These adipose-derived signals may also disturb hypothalamic–pituitary–thyroid (HPT) axis regulation.

An emerging concept is the gut–liver–thyroid axis. Alterations in gut microbiota composition, intestinal permeability, and bile acid metabolism—frequent in obese adolescents—may influence both hepatic lipid handling and thyroid function (41). Microbial metabolites such as short-chain fatty acids and secondary bile acids can modulate metabolic and endocrine pathways (42). Although pediatric data are limited, this axis provides a plausible mechanistic framework for the co-occurrence of MAFLD and SCH and may represent a novel therapeutic target (43).

Together, these pathophysiological mechanisms underscore a multifactorial and bidirectional relationship between SCH and MAFLD in adolescents. Rather than isolated entities, the two conditions appear to converge at the nexus of hormonal regulation, metabolic signaling, and inflammatory activation. This evolving understanding highlights the need for integrated diagnostic and therapeutic strategies that consider the endocrine-liver interface in managing metabolic disorders in youth (Table 1).

**Table 1:** Pathophysiological mechanisms linking subclinical hypothyroidism and MAFLD in adolescents.

Pathophysiological module	Key pathways	Impact on MAFLD	Impact on SCH
TSH and hepatic lipid metabolism (6)	TSH → TSHR → SREBP-1c ↑ → lipogenesis ↑	Promotes hepatic lipid	Sustained TSH elevation
Insulin resistance (37)	↓T3→↓GLUT expression →↑insulin resistance	Exacerbates hepatic metabolic dysfunction	Impaired T3 metabolic activity
Chronic low-grade inflammation (38)	↑TNF-α/↑IL-6→liver injury & thyroid hormone resistance	Activates inflammation and hepatocyte injury	Inflammation disrupts thyroid hormone signaling
Adipokine imbalance (38, 40)	↑Leptin/↑Resistin/↓Adiponectin → metabolic dysfunction	Increases lipogenesis and insulin	Dysregulation of the HPT axis
Gut–liver–thyroid axis (41)	SCFA/bile acid dysregulation→liver and thyroid function affected	Microbiota-derived signals promote steatosis	Microbiota affects TSH regulation and hormone

## Evidence from recent studies

A growing body of clinical evidence supports the association between SCH and MAFLD in adolescents. Although most available studies are cross-sectional or retrospective in design, their findings consistently suggest that thyroid dysfunction, particularly elevated TSH levels, is linked to hepatic steatosis and metabolic disturbances in obese youth.

Several observational studies have reported a higher prevalence of SCH among adolescents with MAFLD, and vice versa. In a multicenter retrospective study conducted by Choi et al. (44) involving 428 Korean children with NAFLD, 13.6% were diagnosed with SCH. The prevalence of SCH increased progressively with liver disease severity, from 1.1% in mild steatosis to 55.4% in severe cases. Multivariate analysis demonstrated that severe steatosis (OR = 98.35, 95% CI: 22.4–431.9) and elevated aspartate aminotransferase-to-platelet ratio index (APRI) were independent predictors of SCH. These findings highlight a possible dose-response relationship between hepatic fat accumulation and thyroid dysfunction. Similarly, Xue et al. (45) examined 141 obese boys and found that those with mild SCH (*n* = 47) exhibited a higher prevalence of NAFLD and metabolic syndrome compared to euthyroid counterparts. They also had lower IGF-1 SDS scores and higher BMI SDS, with multivariate models confirming both as independent predictors of SCH. These results underscore a potential link between impaired growth factor signaling, adiposity, and thyroid dysfunction.

Longitudinal insights are limited but emerging. In a retrospective cohort study by Untalan et al. (46), SCH was significantly more common in adolescents with imaging- or biopsy-confirmed MAFLD. Moreover, persistent TSH elevation over time was associated with histological progression of liver disease, including worsening steatosis and fibrosis. These findings suggest that SCH may not only coexist with MAFLD but also influence its natural history. Beyond associations, attempts have been made to explore causality using statistical mediation analysis. A study applying causal mediation modeling (47) among children with biopsy-proven NAFLD revealed that TSH in the highest quartile (>2.35 mIU/L) conferred a 4.6-fold increased odds of NAFLD. Importantly, TSH accounted for approximately 34% of the total effect of modified BMI-z on NAFLD risk, with 16% mediated indirectly through TSH and 18% reflecting a direct effect independent of obesity. These findings imply that TSH may serve as a partial mediator between obesity and fatty liver, rather than being a mere bystander.

Narrative reviews have further reinforced the biological plausibility of a bidirectional relationship between SCH and MAFLD in adolescents. For example, Calcaterra et al. (39) proposed a feedback model in which obesity-driven inflammation induces SCH, while elevated TSH promotes hepatic lipogenesis and insulin resistance. Kaltenbach et al. (48) also reported that among 332 obese children, those with hepatic steatosis had significantly higher TSH levels. Additional studies have observed elevated levels of total and free triiodothyronine (T3) with concomitant reductions in FT4 in MAFLD patients, potentially reflecting tissue-level hypothyroidism and adaptive thyroidal responses to metabolic stress.

Although most existing data derive from observational studies, preliminary interventional trials in adults offer additional support for a causal link. A *post hoc* analysis of a randomized controlled trial by Liu et al. (49) showed that levothyroxine (LT4) therapy significantly reduced NAFLD prevalence in adults with significant SCH (from 48.5% to 24.2%, *p* = 0.041), and improved liver enzymes. In mild SCH patients with dyslipidemia, LT4 supplementation led to modest improvements in hepatic and metabolic parameters. Consistent findings were reported by Mahran et al. (50) in a prospective RCT, where adult patients with SCH and MASLD showed significant improvements in liver fat content, steatosis grades (by ultrasound and CAP), liver enzymes, BMI, and lipid profiles following LT4 treatment. For example, MASLD prevalence declined to 50% in the significant SCH-LT4 group (*p* = 0.001) and to 60% in the mild SCH-LT4 group (*p* = 0.025), while the untreated group showed only non-significant improvement.

Collectively, these findings support a consistent association between SCH and MAFLD in adolescents, with emerging evidence—both mechanistic and interventional—suggesting a potential causal pathway mediated through TSH. However, the heterogeneity of study designs, populations, and diagnostic criteria limits the strength of inference. Well-powered, prospective studies and pediatric-specific randomized trials are needed to confirm temporality and elucidate whether targeting thyroid dysfunction may alter the trajectory of pediatric fatty liver disease (Table 2).

**Table 2:** Summary of studies exploring the association between subclinical hypothyroidism and MAFLD in adolescents.

Study	Sample size	Design	Key findings
Choi et al., (44)	428 children	Multicenter retrospective cross-sectional	SCH prevalence rose with steatosis severity (1.1% to 55.4%); severe steatosis and APRI were independent predictors
Xue et al., (45)	141 obese boys	Single-center cross-sectional	Mild SCH associated with NAFLD, MetS; lower IGF-1 SDS and higher BMI SDS were independent predictors
Untalan et al., (46)	Not specified; adolescents with biopsy/imaging-confirmed MAFLD	Retrospective cohort	Persistent TSH elevation associated with histologic progression (steatosis, fibrosis)
Causal mediation modeling	4,133 children (66 NAFLD cases, 4,067 controls)	Causal mediation analysis (case-control)	TSH Q4 (>2.35 mIU/L) conferred 4.6× NAFLD risk; 34% of BMI-z effect mediated through TSH
Calcaterra et al., (39)	Narrative review	Review	Proposed feedback loop: obesity-induced inflammation causes SCH; TSH promotes hepatic lipogenesis and IR
Kaltenbach et al., (48)	332 obese children	Single-center cross-sectional	Children with steatosis had significantly higher TSH levels; TSH independently associated with NAFLD
Liu et al., (49)	363 adults with SCH	Post hoc RCT analysis	LT4 reduced NAFLD prevalence (48.5% to 24.2%) and improved liver enzymes
Mahran et al., (50)	182 adults with SCH + MASLD	Prospective RCT	LT4 significantly improved liver fat, BMI, lipids; MASLD declined to 50%–60% post-treatment

## Screening, risk assessment, and treatment implications

Given the emerging evidence linking SCH to MAFLD in adolescents, there is a compelling rationale for integrated screening strategies in high-risk populations. Routine evaluation of thyroid function—particularly measurement of serum TSH and FT4—may offer additional clinical insight in adolescents with hepatic steatosis, especially those with obesity, insulin resistance, or abnormal liver enzymes. Similarly, adolescents diagnosed with SCH, even in the absence of overt hypothyroid symptoms, should be considered for hepatic assessment using non-invasive tools such as abdominal ultrasonography or transient elastography. Early identification of concurrent liver and thyroid dysfunction could allow for timely lifestyle or pharmacological interventions aimed at preventing long-term metabolic complications.

TSH levels may also serve as surrogate biomarkers of systemic metabolic stress and hepatic vulnerability. Incorporating thyroid function tests into standard metabolic screening panels for adolescents with obesity or features of the metabolic syndrome may enhance risk stratification. Furthermore, monitoring longitudinal trends in TSH could aid in predicting progression or resolution of hepatic steatosis, particularly in patients undergoing weight management or pharmacologic therapy.

Despite increasing recognition of the SCH–MAFLD interplay, the optimal management of adolescents with both conditions remains uncertain. Current guidelines generally recommend a conservative approach to SCH, with treatment typically reserved for individuals with TSH >10 mIU/L, overt hypothyroidism, or significant symptom burden (51). However, in the context of MAFLD, this threshold may warrant reevaluation. If elevated TSH actively contributes to hepatic lipid accumulation, as suggested by mechanistic and mediation studies, targeted thyroid hormone therapy—such as low-dose levothyroxine—could offer hepatic as well as metabolic benefit (52). Preliminary adult studies have reported improvements in liver fat content and aminotransferase levels following normalization of TSH with levothyroxine (49, 50, 53). While pediatric data are limited, such findings support the need for clinical trials assessing whether thyroid-directed treatment can modify the course of MAFLD in adolescents with persistent SCH (39). Importantly, any pharmacological intervention should be balanced against the potential for overtreatment, especially given the fluctuating and often transient nature of SCH in youth.

In parallel, lifestyle modification remains the cornerstone of management for both conditions. Caloric restriction, increased physical activity, and reduction in screen time have all been shown to improve both thyroid function and hepatic steatosis in adolescents. Even modest weight loss can lead to reductions in TSH levels and improvements in liver fat content (54). These findings reinforce the value of comprehensive lifestyle programs as first-line therapy, with potential for dual endocrine and hepatic benefit.

From a public health perspective, awareness of the endocrine–hepatic connection in pediatric obesity is essential. Clinicians should be alert to subtle endocrine abnormalities that may signal increased risk for liver disease, while educational efforts targeting families and schools should emphasize the systemic consequences of childhood metabolic dysfunction. Establishing integrated care pathways that include or liver assessment for adolescents with SCH (38).

## Future research directions

Although evidence increasingly hints at a connection betweencc SCH and MAFLD in adolescents, many aspects remain unresolved. The field still lacks studies that can unravel not only if the two conditions are related, but also how and in what order they develop.

A logical next step would be to follow adolescents over several years, tracking thyroid hormone levels, liver fat content, and key metabolic markers at multiple time points. Such longitudinal designs could help distinguish whether thyroid dysfunction emerges before, alongside, or after hepatic steatosis. These studies would also need to account for important modifiers—pubertal stage, sex steroid levels, and genetic predisposition—so as to avoid spurious associations. In some cases, genetic approaches like Mendelian randomization might help to test whether a lifelong tendency toward higher TSH is inherently tied to greater liver fat accumulation.

Another issue worth addressing is whether intervention can change the course of disease. Could correcting thyroid function—using levothyroxine or other targeted strategies—translate into measurable improvements in liver health? Randomized trials could explore this, looking at outcomes such as reduced hepatic fat fraction on MRI, lower aminotransferase concentrations, or improvements in non-invasive fibrosis scores. It would also be valuable to see whether pairing thyroid treatment with lifestyle modification or insulin-sensitizing agents yields better results than either option alone.

Until such evidence is available, any firm recommendations on screening or treatment in this young population would be premature. The answers, however, will be critical for shaping prevention and management strategies in a group that stands to benefit from early and effective intervention.

## Mechanistic and translational research

Understanding how thyroid dysfunction shapes hepatic lipid metabolism in adolescents requires bridging mechanistic research with translational applications. Recent work indicates that TSH may directly activate hepatocyte receptors, influencing key regulators such as SREBP-1c, PPAR-γ, and AMPK, which collectively govern lipid synthesis, oxidation, and storage. Thyroid hormones also affect mitochondrial biogenesis and redox balance, potentially altering vulnerability to oxidative stress and lipotoxic injury. In addition, metabolic tissues may act as an interface where adipokines, inflammatory mediators, and thyroid hormone receptors converge, although the sequence and relative importance of these interactions remain to be established. To capture adolescent-specific physiology, experimental models such as induced pluripotent stem cell–derived hepatocytes and juvenile animal models should be prioritized over adult-based systems.

An emerging but underexplored area is the gut–liver–thyroid axis, which integrates microbial, hepatic, and endocrine signaling. Evidence from adult and limited pediatric studies suggests that microbial metabolites—such as short-chain fatty acids, secondary bile acids, and lipopolysaccharides—can modulate both hepatic steatosis and thyroid hormone activity (55, 56). Given that adolescence is a period of dynamic gut microbiota development, disruptions in this axis could have unique consequences for metabolic health. Future studies should combine metagenomic sequencing, metabolomic profiling, and intestinal permeability assays to define microbial and metabolic signatures associated with concurrent SCH and MAFLD in youth. Interventional approaches, including probiotics, prebiotics, or dietary fiber supplementation, could then test whether modulation of the gut–liver–thyroid axis can improve both hepatic and thyroid outcomes.

Finally, the absence of pediatric-specific clinical guidelines poses a major limitation for practice. Current thresholds for diagnosing and treating SCH are largely extrapolated from adult populations and may not reflect developmental and pubertal variations in TSH physiology. Research should aim to determine age-appropriate TSH cut-offs that correlate with increased liver risk, establish evidence-based screening intervals for thyroid function in adolescents with obesity or hepatic steatosis, and clarify when levothyroxine therapy is warranted in the setting of hepatic involvement. Such efforts would lay the groundwork for precision screening and personalized interventions that address the complex interplay between endocrine and hepatic health in adolescence.
