# Supplementary material for: Exploring the link between metabolic dysfunction-associated fatty liver disease and subclinical hypothyroidism in adolescents: a comprehensive review
Source: Front Pediatr. 2026 Feb 16;14:1696331. doi: 10.3389/fped.2026.1696331 (PMC12950748; doi:10.3389/fped.2026.1696331)
Supplement: Supplementary file 1 [file Table1.docx]

| **Study (first author, year)** | **Design** | **Tool (NOS version)** | **Selection (0–4★)** | **Comparability (0–2★)** | **Exposure/Outcome (0–3★)** | **Total (0–9★)** | **Overall risk of bias (qualitative)** | **Key considerations** |
| --- | --- | --- | --- | --- | --- | --- | --- | --- |
| Nichols 2020 | Case–control (biopsy-proven NAFLD cases vs controls) | NOS – case–control | ★★★★ | ★★ | ★★☆ | **8/9** | Low to moderate | Clear case definition (biopsy-proven NAFLD), appropriate controls from same source population, standardized laboratory assessment of TSH for cases/controls, key confounders (age, sex, obesity severity) adjusted. Non-response and selection processes for controls not fully detailed. |
| Untalan 2024 | Prospective / nested cohort (NASH CRN trials + NHANES controls) | NOS – cohort | ★★★★ | ★★ | ★★★ | **9/9** | Low | MASLD cohort and population controls well-defined; exposure (TSH) and outcomes (histology, MASLD features) measured with validated methods; key confounders (age, sex, race/ethnicity, BMI) adjusted; longitudinal follow-up and attrition adequately reported. |

NOS ratings were applied using design-appropriate versions (case–control vs cohort). Stars reflect domain-level judgments based on the published information; we did not impose arbitrary cut-offs to label studies as “high” or “low” quality but used totals descriptively.
