# Supplementary material for: Exploring the link between metabolic dysfunction-associated fatty liver disease and subclinical hypothyroidism in adolescents: a comprehensive review
Source: Front Pediatr. 2026 Feb 16;14:1696331. doi: 10.3389/fped.2026.1696331 (PMC12950748; doi:10.3389/fped.2026.1696331)
Supplement: Supplementary file 2 [file Table2.docx]

| **Study (first author, year)** | **Design** | **Tool** | **Q1** | **Q2** | **Q3** | **Q4** | **Q5** | **Q6** | **Q7** | **Q8** | **Summary judgment** | **Key considerations** |
| --- | --- | --- | --- | --- | --- | --- | --- | --- | --- | --- | --- | --- |
| Choi 2021 | Multicenter analytical cross-sectional (pediatric NAFLD + SH) | JBI cross-sectional | Yes | Yes | Yes | Yes | Yes | Yes | Yes | Yes | Low risk | Clear inclusion criteria; pediatric NAFLD defined by imaging/non-invasive fibrosis; TSH/SH defined using standard cut-offs; important confounders (age, sex, BMI, fibrosis/steatosis grade) considered with multivariable models; appropriate regression analyses. |
| Kaltenbach 2017 | Analytical cross-sectional (overweight/obese children) | JBI cross-sectional | Yes | Yes | Yes | Yes | Yes | Yes | Yes | Yes | Low risk | Well-described overweight clinic cohort; standardized ultrasound for steatosis; TSH measured in accredited lab; confounders (age, BMI-SDS, puberty) identified and included in multivariable analyses; appropriate statistics. |
| Xue 2025 | Analytical cross-sectional (obese boys with/without mild SCH) | JBI cross-sectional | Yes | Yes | Yes | Yes | Yes | Partial | Yes | Yes | Low to moderate | Clear inclusion and exposure definition (MSH vs non-MSH); NAFLD and MS defined; confounders (BMI SDS, metabolic parameters) discussed; some adjustment strategies reported but residual confounding (diet, activity, puberty stage) possible; overall appropriate analytic approach. |

The JBI analytical cross-sectional checklist (8 items) was applied to each study. Items were rated Yes/No/Partial based on reported methods. We summarize overall risk qualitatively without converting scores into arbitrary pass/fail thresholds.
