# Supplementary figures and images for: Exploring the link between metabolic dysfunction-associated fatty liver disease and subclinical hypothyroidism in adolescents: a comprehensive review
Source: Front Pediatr. 2026 Feb 16;14:1696331. doi: 10.3389/fped.2026.1696331 (PMC12950748; doi:10.3389/fped.2026.1696331)

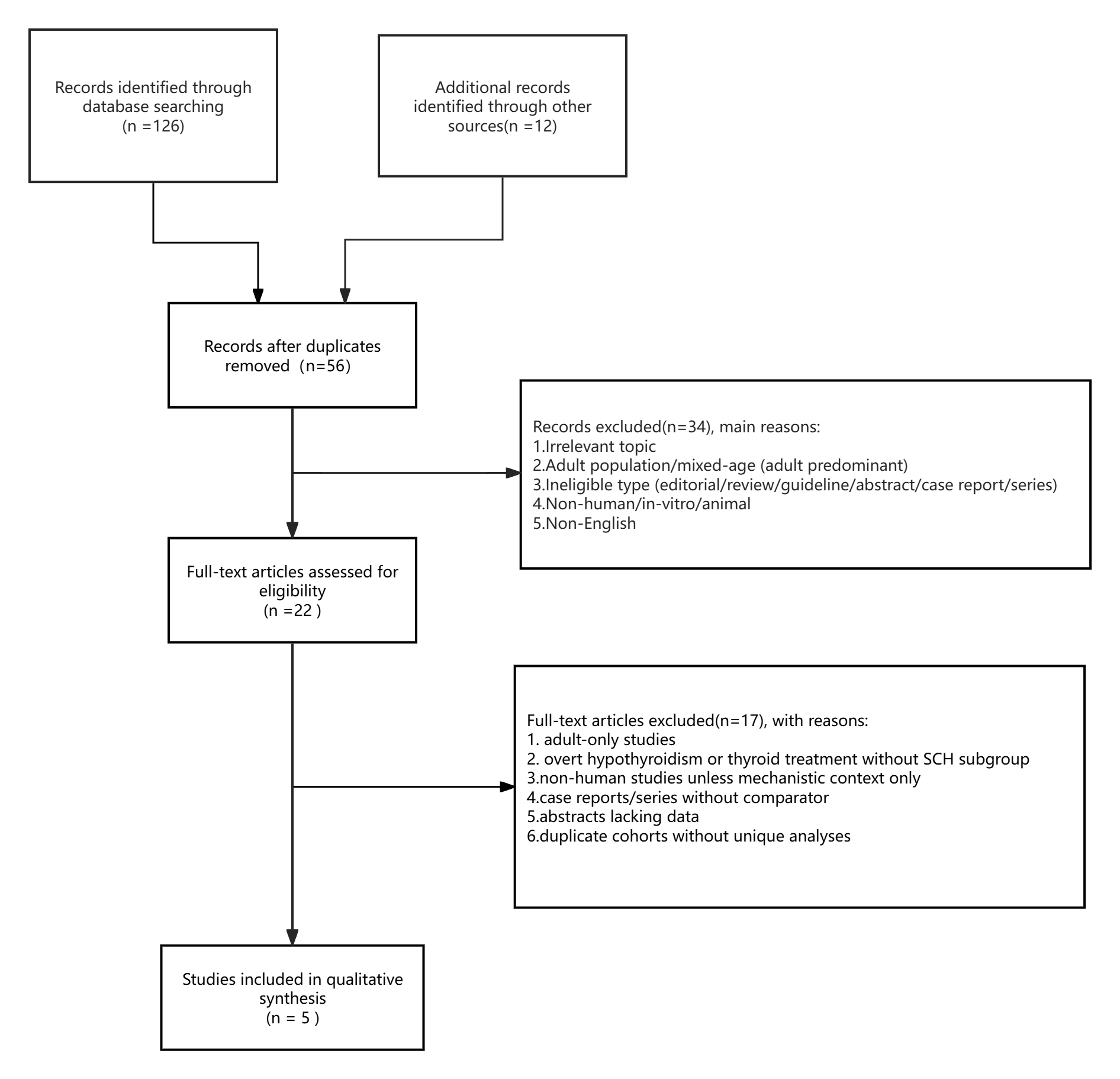

Supplement: Supplementary file 3 [file Image1.png]
